# Series: Practical guidance to qualitative research. Part 2: Context, research questions and designs

**DOI:** 10.1080/13814788.2017.1375090

**Published:** 2017-11-29

**Authors:** Irene Korstjens, Albine Moser

**Affiliations:** a Faculty of Health Care, Research Centre for Midwifery Science, Zuyd University of Applied Sciences, Maastricht, The Netherlands;; b Faculty of Health Care, Research Centre Autonomy and Participation of Chronically Ill People, Zuyd University of Applied Sciences, Heerlen, The Netherlands;; c Faculty of Health, Medicine and Life Sciences, Department of Family Medicine, Maastricht University, Maastricht, The Netherlands

**Keywords:** General practice/family medicine, general qualitative designs and methods

## Abstract

In the course of our supervisory work over the years, we have noticed that qualitative research tends to evoke a lot of questions and worries, so-called frequently asked questions (FAQs). This series of four articles intends to provide novice researchers with practical guidance for conducting high-quality qualitative research in primary care. By ‘novice’ we mean Master’s students and junior researchers, as well as experienced quantitative researchers who are engaging in qualitative research for the first time. This series addresses their questions and provides researchers, readers, reviewers and editors with references to criteria and tools for judging the quality of qualitative research papers. This second article addresses FAQs about context, research questions and designs. Qualitative research takes into account the natural contexts in which individuals or groups function to provide an in-depth understanding of real-world problems. The research questions are generally broad and open to unexpected findings. The choice of a qualitative design primarily depends on the nature of the research problem, the research question(s) and the scientific knowledge one seeks. Ethnography, phenomenology and grounded theory are considered to represent the ‘big three’ qualitative approaches. Theory guides the researcher through the research process by providing a ‘lens’ to look at the phenomenon under study. Since qualitative researchers and the participants of their studies interact in a social process, researchers influence the research process. The first article described the key features of qualitative research, the third article will focus on sampling, data collection and analysis, while the last article focuses on trustworthiness and publishing.

Key points on context, research questions and designs

- Research questions are generally, broad and open to unexpected findings, and depending on the research process might change to some extent.
- The SPIDER tool is more suited than PICO for searching for qualitative studies in the literature, and can support the process of formulating research questions for original studies.
- The choice of a qualitative design primarily depends on the nature of the research problem, the research question, and the scientific knowledge one seeks.
- Theory guides the researcher through the research process by providing a ‘lens’ to look at the phenomenon under study.
- Since qualitative researchers and the participants interact in a social process, the researcher influences the research process.

## Introduction

In an introductory paper [1], we have described the key features of qualitative research. The current article addresses frequently asked questions about context, research questions and design of qualitative research.

## Context

### Why is context important?

Qualitative research takes into account the natural contexts in which individuals or groups function, as its aim is to provide an in-depth understanding of real-world problems [2]. In contrast to quantitative research, generalizability is not a guiding principle. According to most qualitative researchers, the ‘reality’ we perceive is constructed by our social, cultural, historical and individual contexts. Therefore, you look for variety in people to describe, explore or explain phenomena in real-world contexts. Influence from the researcher on the context is inevitable. However, by striving to minimalize your interfering with people’s natural settings, you can get a ‘behind the scenes’ picture of how people feel or what other forces are at work, which may not be discovered in a quantitative investigation. Understanding what practitioners and patients think, feel or do in their natural context, can make clinical practice and evidence-based interventions more effective, efficient, equitable and humane. For example, despite their awareness of widespread family violence, general practitioners (GPs) seem to be hesitant to ask about intimate partner violence. By applying a qualitative research approach, you might explore how and why practitioners act this way. You need to understand their context to be able to interact effectively with them, to analyse the data, and report your findings. You might consider the characteristics of practitioners and patients, such as their age, marital status, education, health condition, physical environment or social circumstances, and how and where you conduct your observations, interviews and group discussions. By giving your readers a ‘thick description’ of the participants’ contexts you render their behaviour, experiences, perceptions and feelings meaningful. Moreover, you enable your readers to consider whether and how the findings of your study can be transferred to their contexts.

## Research questions

### Why should the research question be broad and open?

To enable a thorough in-depth description, exploration or explanation of the phenomenon under study, in general, research questions need to be broad and open to unexpected findings. Within more in-depth research, for example, during building theory in a grounded theory design, the research question might be more focused. Where quantitative research asks: ‘how many, how much, and how often?’ qualitative research would ask: ‘what?’ and even more ‘how, and why?’ Depending on the research process, you might feel a need for fine-tuning or additional questions. This is common in qualitative research as it works with ‘emerging design,’ which means that it is not possible to plan the research in detail at the start, as the researchers have to be responsive to what they find as the research proceeds. This flexibility within the design is seen as a strength in qualitative research but only within an overall coherent methodology.

### What kind of literature would I search for when preparing a qualitative study?

You would search for literature that can provide you with insights into the current state of knowledge and the knowledge gap that your study might address (Box 1). You might look for original quantitative, mixed-method and qualitative studies, or reviews such as quantitative meta-analyses or qualitative meta-syntheses. These findings would give you a picture of the empirical knowledge gap and the qualitative research questions that might lead to relevant and new insights and useful theories, models or concepts for studying your topic. When little knowledge is available, a qualitative study can be a useful starting point for subsequent studies. If in preparing your qualitative study, you cannot find sufficient literature about your topic, you might turn to proxy literature to explore the landscape around your topic. For example, when you are one of the very first researchers to study shared decision-making or health literacy in maternity care for disadvantaged parents-to-be, you might search for existing literature on these topics in other healthcare settings, such as general practice.

Box 1. Searching the literature for qualitative studies: the SPIDER tool. Based on Cooke et al. [3].

**Table:** 

S	**Sample**: qualitative research uses smaller samples, as findings are not intended to be generalized to the general population.
PI	**Phenomenon of Interest**: qualitative research examines how and why certain experiences, behaviours and decisions occur (in contrast to the effectiveness of intervention).
D	**Design**: refers to the theoretical framework and the corresponding method used, which influence the robustness of the analysis and findings.
E	**Evaluation**: evaluation outcomes may include more subjective outcomes (views, attitudes, perspectives, experiences, etc.).
R	**Research type**: qualitative, quantitative and mixed-methods research could be searched for.

### Why do qualitative researchers prefer SPIDER to PICO?

The SPIDER tool (sample-phenomenon of interest-design-evaluation-research type) (Box 1) is one of the available tools for qualitative literature searches [3]. It has been specifically developed for qualitative evidence synthesis, making it more suitable than PICO (population-intervention-comparison-outcome) in searching for qualitative studies that focus on understanding real-life experiences and processes of a variety of participants. PICO is primarily a tool for collecting evidence from published *quantitative* research on prognoses, diagnoses and therapies. Quantitative studies mostly use larger samples, comparing intervention and control groups, focusing on quantification of predefined outcomes at group level that can be generalized to larger populations. In contrast, qualitative research studies smaller samples in greater depth; it strives to minimalize manipulating their natural settings and is open to rich and unexpected findings. To suit this approach, the SPIDER tool was developed by adapting the PICO tool. Although these tools are meant for searching the literature, they can also be helpful in formulating research questions for original studies. Using SPIDER might support you in formulating a broad and open qualitative research question.

An example of an SPIDER-type question for a qualitative study using interviews is: ‘What are young parents’ experiences of attending antenatal education?’ The abstract and introduction of a manuscript might contain this broad and open research question, after which the methods section provides further operationalization of the elements of the SPIDER tool, such as (S) young mothers and fathers, aged 17–27 years, 1–12 months after childbirth, low to high educational levels, in urban or semi-urban regions; (PI) experiences of antenatal education in group sessions during pregnancy guided by maternity care professionals; (D) phenomenology, interviews; (E) perceived benefits and costs, psychosocial and peer support received, changes in attitude, expectations, and perceived skills regarding healthy lifestyle, childbirth, parenthood, etc.; and (R) qualitative.

### Is it normal that my research question seems to change during the study?

During the research process, the research question might change to a certain degree because data collection and analysis sharpens the researcher’s lenses. Data collection and analysis are iterative processes that happen simultaneously as the research progresses. This might lead to a somewhat different focus of your research question and to additional questions. However, you cannot radically change your research question because that would mean you were performing a different study. In the methods section, you need to describe how and explain why the original research question was changed.

For example, let us return to the problem that GPs are hesitant to ask about intimate partner violence despite their awareness of family violence. To design a qualitative study, you might use SPIDER to support you in formulating your research question. You purposefully sample GPs, varying in age, gender, years of experience and type of practice (S-1). You might also decide to sample patients, in a variety of life situations, who have been faced with the problem (S-2). You clarify the phenomenon of family violence, which might be broadly defined when you design your study—e.g. family abuse and violence (PI-1). However, as your study evolves you might feel the need for fine-tuning—e.g. asking about intimate partner violence (PI-2). You describe the design, for instance, a phenomenological study using interviews (D), as well as the ‘think, feel or do’ elements you want to evaluate in your qualitative research. Depending on what is already known and the aim of your research, you might choose to describe actual behaviour and experiences (E-1) or explore attitudes and perspectives (E-2). Then, as your study progresses, you also might want to explain communication and follow-up processes (E-3) in your qualitative research (R).

Each of your choices will be a trade-off between the intended variety, depth and richness of your findings and the required samples, methods, techniques and efforts for data collection and analyses. These choices lead to different research questions, for example:

- ‘What are GPs’ and patients’ attitudes and perspectives towards discussing family abuse and violence?’ Or:
- ‘How do GPs behave during the communication and follow-up process when a patient’s signals suggest intimate partner violence?’

## Designing qualitative studies

### How do I choose a qualitative design?

As in quantitative research, you base the choice of a qualitative design primarily on the nature of the research problem, the research question and the scientific knowledge you seek. Therefore, instead of simply choosing what seems easy or interesting, it is wiser to first consider and discuss with other qualitative researchers the pros and cons of different designs for your study. Then, depending on your skills and your knowledge and understanding of qualitative methodology and your research topic, you might seek training or support from other qualitative researchers. Finally, just as in quantitative research, the resources and time available and your access to the study settings and participants also influence the choices you make in designing the study.

### What are the most important qualitative designs?

Ethnography [4], phenomenology [5], and grounded theory [6] are considered the ‘big three’ qualitative approaches [7] (Box 2). Box 2 shows that they stem from different theoretical disciplines and are used in various domains focusing on different areas of inquiry. Furthermore, qualitative research has a rich tradition of various designs [2]. Box 3 presents other qualitative approaches such as case studies [8], conversation analysis [9], narrative research [10], hermeneutic research [11], historical research [12], participatory action research and [13], participatory community research [14], and research based on critical social theory [15], for example, feminist research or empowerment evaluation [16]. Some researchers do not mention a specific qualitative approach or research tradition but use a descriptive generic research [17] or say that they used thematic analysis or content analysis, an analysis of themes and patterns that emerge in the narrative content from a qualitative study [2]. This form of data analysis will be addressed in Part 3 of our series.

Box 2. The ‘big three’ approaches in qualitative study design. Based on Polit and Beck [2].

**Table:** 

	Ethnography	Phenomenology	Grounded theory
Definition	A branch of human enquiry, associated with anthropology that focuses on the culture of a group of people, with an effort to understand the world view of those under study.	A qualitative research tradition, with roots in philosophy and psychology, that focuses on the lived experience of humans.	A qualitative research methodology with roots in sociology that aims to develop theories grounded in real-world observations.
Discipline	Anthropology	Psychology, philosophy	Sociology
Domain	Culture	Lived experience	Social settings
Area of inquiry	Holistic view of a culture.	Experiences of individuals within their experiential world or ‘life-world’.	Social structural process within a social setting.
Focus	Understanding the meanings and behaviours associated with the membership of groups, teams, etc.	Exploring how individuals make sense of the world to provide insightful accounts of their subjective experience.	Building theories about social phenomena.

Box 3. Definitions of other qualitative research approaches. Based on Polit and Beck [2].

**Table:** 

Case study	A research method involving a thorough, in-depth analysis of an individual, group or other social unit.
Conversation analysis	Form of discourse analysis, a qualitative tradition from the discipline of sociolinguistics that seeks to understand the rules, mechanisms, and structure of conversations.
Critical social theory	An approach to viewing the world that involves a critique of society, with the goal of envisioning new possibilities and effecting social change.
Feminist research	Research that seeks to understand, typically through qualitative approaches, how gender and a gendered social order shape women’s lives and their consciousness.
Hermeneutics	A qualitative research tradition, drawing on interpretative phenomenology that focuses on the lived experience of humans, and how they interpret those experiences.
Historical research	Systematic studies designed to discover facts and relationships about past events.
Narrative research	A narrative approach that focuses on the story as the object of the inquiry.
Participatory action research	A collaborative research approach between researchers and participants based on the premise that the production of knowledge can be political and used to exert power.
Community-based participatory research	A research approach that enlists those who are most affected by a community issue—typically in collaboration or partnership with others who have research skills—to conduct research on and analyse that issue, with the goal to resolve it.
Content analysis	The process or organizing and integrating material from documents, often-narrative information from a qualitative study, according to key concepts and themes.

### Depending on your research question, you might choose one of the ‘big three’ designs

Let us assume that you want to study the caring relationship in palliative care in a primary care setting for people with COPD. If you are interested in the care provided by family caregivers from different ethnic backgrounds, you will want to investigate their experiences. Your research question might be ‘What constitutes the caring relationship between GPs and family caregivers in the palliative care for people with COPD among family caregivers of Moroccan, Syrian, and Iranian ethnicity?’ Since you are interested in the caring relationship within cultural groups or subgroups, you might choose ethnography. Ethnography is the study of culture within a society, focusing on one or more groups. Data is collected mostly through observations, informal (ethnographic) conversations, interviews and/or artefacts. The findings are presented in a lengthy monograph where concepts and patterns are presented in a holistic way using context-rich description.

If you are interested in the experiential world or ‘life-world’ of the family caregivers and the impact of caregiving on their own lives, your research question might be ‘What is the lived experience of being a family caregiver for a family member with COPD whose end is near?’ In such a case, you might choose phenomenology, in which data are collected through in-depth interviews. The findings are presented in detailed descriptions of participants’ experiences, grouped in themes.

If you want to study the interaction between GPs and family caregivers to generate a theory of ‘trust’ within caring relationships, your research question might be ‘How does a relationship of trust between GPs and family caregivers evolve in end-of-life care for people with COPD?’ Grounded theory might then be the design of the first choice. In this approach, data are collected mostly through in-depth interviews, but may also include observations of encounters, followed by interviews with those who were observed. The findings presented consist of a theory, including a basic social process and relevant concepts and categories.

If you merely aim to give a qualitative description of the views of family caregivers about facilitators and barriers to contacting GPs, you might use *content analysis* and present the themes and subthemes you found.

### What is the role of theory in qualitative research?

The role of theory is to guide you through the research process. Theory supports formulating the research question, guides data collection and analysis, and offers possible explanations of underlying causes of or influences on phenomena. From the start of your research, theory provides you with a ‘lens’ to look at the phenomenon under study. During your study, this ‘theoretical lens’ helps to focus your attention on specific aspects of the data and provides you with a conceptual model or framework for analysing them. It supports you in moving beyond the individual ‘stories’ of the participants. This leads to a broader understanding of the phenomenon of study and a wider applicability and transferability of the findings, which might help you formulate new theory, or advance a model or framework. Note that research does not need to be always theory-based, for example, in a descriptive study, interviewing people about perceived facilitators and barriers for adopting new behaviour.

### What is my role as a researcher?

As a qualitative researcher, you influence the research process. Qualitative researchers and the study participants always interact in a social process. You build a relationship midst data collection, for the short-term in an interview, or for the long-term during observations or longitudinal studies. This influences the research process and its findings, which is why your report needs to be transparent about your perspective and explicitly acknowledge your subjectivity. Your role as a qualitative researcher requires empathy as well as distance. By empathy, we mean that you can put yourself into the participants’ situation. Empathy is needed to establish a trusting relationship but might also bring about emotional distress. By distance, we mean that you need to be aware of your values, which influence your data collection, and that you have to be non-judgemental and non-directive.

There is always a power difference between the researcher and participants. Especially, feminist researchers acknowledge that the research is done by, for, and about women and the focus is on gender domination and discrimination. As a feminist researcher, you would try to establish a trustworthy and non-exploitative relationship and place yourself within the study to avoid objectification. Feminist research is transformative to change oppressive structures for women [16].

### What ethical issues do I need to consider?

Although qualitative researchers do not aim to intervene, their interaction with participants requires careful adherence to the statement of ethical principles for medical research involving human subjects as laid down in the Declaration of Helsinki [18]. It states that healthcare professionals involved in medical research are obliged to protect the life, health, dignity, integrity, right to self-determination, privacy and confidentiality of personal information of research subjects. The Declaration also warrants that all vulnerable groups and individuals should receive specifically considered protection. This is also relevant when working in contexts of low-income countries and poverty. Furthermore, researchers must consider the ethical, legal and regulatory norms and standards in their own countries, as well as applicable international norms and standards. You might contact your local Medical Ethics Committee before setting up your study. In some countries, Medical Ethics Committees do not review qualitative research [2]. In that case, you will have to adhere to the Declaration of Helsinki [18], and you might seek approval from a research committee at your institution or the board of your institution.

In qualitative research, you have to ensure anonymity by code numbering the tapes and transcripts and removing any identifying information from the transcripts. When you work with transcription offices, they will need to sign a confidentiality agreement. Even though the quotes from participants in your manuscripts are anonymized, you cannot always guarantee full confidentiality. Therefore, you might ask participants special permission for using these quotes in scientific publications.

The next article in this Series on qualitative research, Part 3, will focus on sampling, data collection, and analysis [19]. In the final article, Part 4, we address two overarching themes: trustworthiness and publishing [20].
